# Clinical Significance of Two Real-Time PCR Assays for Chronic Hepatitis C Patients Receiving Protease Inhibitor-Based Therapy

**DOI:** 10.1371/journal.pone.0170667

**Published:** 2017-01-24

**Authors:** Takako Inoue, Su Su Hmwe, Noritomo Shimada, Keizo Kato, Tatsuya Ide, Takuji Torimura, Takashi Kumada, Hidenori Toyoda, Akihito Tsubota, Koichi Takaguchi, Takaji Wakita, Yasuhito Tanaka

**Affiliations:** 1 Department of Clinical Laboratory, Nagoya City University Hospital, Nagoya, Japan; 2 Department of Virology II, National Institute of Infectious Diseases, Tokyo, Japan; 3 Ootakanomori Hospital, Kashiwa, Japan; 4 Division of Gastroenterology and Hepatology, Shin-Matsudo Central General Hospital, Matsudo, Japan; 5 Division of Gastroenterology, Department of Medicine, Kurume University School of Medicine, Kurume, Japan; 6 Department of Gastroenterology and Hepatology, Ogaki Municipal Hospital, Ogaki, Japan; 7 Institute of Clinical Medicine and Research, The Jikei University School of Medicine, Kashiwa, Japan; 8 Department of Hepatology, Kagawa Prefectural Central Hospital, Takamatsu, Japan; 9 Department of Virology and Liver Unit, Nagoya City University Graduate School of Medical Sciences, Nagoya, Japan; Saint Louis University, UNITED STATES

## Abstract

The aim of this study was to determine the efficacy of two hepatitis C virus (HCV) real-time PCR assays, the COBAS AmpliPrep/COBAS TaqMan HCV test (CAP/CTM) and the Abbott RealTime HCV test (ART), for predicting the clinical outcomes of patients infected with HCV who received telaprevir (TVR)-based triple therapy or daclatasvir/asunaprevir (DCV/ASV) dual therapy. The rapid virological response rates in patients receiving TVR-based triple therapy were 92% (23/25) and 40% (10/25) for CAP/CTM and ART, respectively. The false omission rate (FOR) of ART was 93.3% (14/15), indicating that CAP/CTM could accurately predict clinical outcome in the early phase. In an independent examination of 20 patients receiving TVR-based triple therapy who developed viral breakthrough or relapse, the times to HCV disappearance by ART were longer than by CAP/CTM, whereas the times to HCV reappearance were similar. In an independent experiment of WHO standard HCV RNA serially diluted in serum containing TVR, the analytical sensitivities of CAP/CTM and ART were similar. However, cell cultures transfected with HCV and grown in medium containing TVR demonstrated that ART detected HCV RNA for a longer time than CAP/CTM. Similar results were found for 42 patients receiving DCV/ASV dual therapy. The FOR of ART was 73.3% (11/15) at week 8 after initiation of therapy, indicating that ART at week 8 could not accurately predict the clinical outcome. In conclusion, although CAP/CTM and ART detected HCV RNA with comparable analytical sensitivity, CAP/CTM might be preferable for predicting the clinical outcomes of patients receiving protease inhibitor-based therapy.

## Introduction

Hepatitis C virus (HCV) affects 130–210 million people worldwide, and HCV infection is a major risk factor for hepatocellular carcinoma [1]. The management of chronic hepatitis C has dramatically improved over the last 5 years, with the time to eradication of HCV becoming shorter [2] [3]. In 2011, the nonstructural (NS) 3/4A protease inhibitor telaprevir (TVR) was approved for clinical use. TVR used with pegylated interferon-α plus ribavirin (Peg-IFN/RBV) has increased the sustained virological response (SVR) rate to greater than 70% [4] [5] [6] [7].

Single nucleotide polymorphisms (SNPs) near the interleukin 28B gene (*IL28B*) of HCV genotype 1 are strongly associated with the null virological response (NVR) of patients receiving Peg-IFN/RBV [8]. Among patients with genotype 1b chronic hepatitis C receiving TVR with Peg-IFN α/RBV (TVR-based triple therapy), the SVR rate of patients with the *IL28B* rs8099917 TT allele was significantly higher than the SVR rate of patients with the *IL28B* TG/GG allele [7]. In 2014, dual therapy with daclatasvir (DCV), a pan-genotypic NS protein 5A inhibitor, and asunaprevir (ASV), an NS3 protease inhibitor, which achieved a high SVR rate for HCV genotype 1 infections, was approved in Japan as the first IFN-free regimen [9].

Quantification of HCV RNA is essential for monitoring chronic hepatitis C treatment and is recommended by the American, European, and Asian-Pacific clinical practice guidelines [10] [11] [12]. Two commercial real-time PCR assays for HCV are available, the Abbott RealTime HCV test (ART; Abbott Diagnostics, Lake Forest, IL, USA) and the COBAS AmpliPrep/COBAS TaqMan HCV test (CAP/CTM; Roche Molecular Systems, Inc., Pleasanton, CA, USA). Both ART and CAP/CTM can accurately predict the clinical outcomes of patients infected with HCV who received Peg-IFN-α2b/RBV [13] [14]. There have also been recent reports on CAP/CTM and ART for evaluating patients receiving TVR-based triple therapy [15] [16]. Although ART was better at detecting HCV RNA, in practice, CAP/CTM may be better at predicting non-SVR [15]. Another study found that ART assays performed at week 4 of treatment showed a higher positive predictive value (PPV) for the achievement of SVR at 24 weeks (SVR24) in patients receiving Peg-IFN α2a/RBV plus the protease inhibitors boceprevir or TVR than CAP/CTM [16]. Additionally, there has been a report on CAP/CTM and ART for evaluating patients receiving triple therapy with the protease inhibitors TVR or simeprevir. The study found that ART can detect lower levels of HCV RNA in the early period than CAP/CTM, and relapse in the late period [17]. Although DCV/ASV dual therapy has now been used in Japan for more than 1 year, to the best of our knowledge, there are no reports of studies comparing CAP/CTM and ART for the evaluation of patients receiving DCV/ASV dual therapy.

We developed the following 3 hypotheses: 1) ART is more sensitive than CAP/CTM for the detection of HCV RNA in serum specimens; 2) TVR inhibits amplification of HCV RNA by CAP/CTM; and 3) small noninfectious HCV RNA fragments remaining in the sera of patients who have resolved HCV viremia are easily detected by ART. In this study, we compared the clinical and analytical sensitivity of CAP/CTM and ART and investigated whether they accurately predicted the clinical outcomes of patients with chronic hepatitis C who were receiving TVR-based triple therapy or DCV/ASV dual therapy.

## Materials and Methods

### Ethical standards

Written informed consent was obtained from each patient, and the study was approved by the local ethics committee of Nagoya City University in accordance with the Declaration of Helsinki (acceptance number: 866).

### Patients

First, a total of 25 patients with chronic HCV genotype 1 infections at Nagoya City University Hospital (Aichi, Japan) were enrolled. All patients started TVR-based triple therapy from November 2011 to May 2013. Prior to starting therapy, all patients treated with TVR-based triple therapy were confirmed by a previously published method [8] to have infections with HCV containing the *IL28B* rs8099917 TT allele. Therefore, these patients were predicted to obtain an SVR to therapy [7] [8].

Second, a total of 42 patients with chronic HCV genotype 1 infections at Nagoya City University Hospital were enrolled. All patients started DCV/ASV dual therapy from September 2014 to April 2015. Both treatment-naïve and treatment-experienced patients were included in this category. Patients were designated treatment naïve if they had never received any anti-HCV therapy for their infection. Patients who had already received one or more types of anti-HCV therapy were designated treatment experienced. The numbers of patients in each subgroup are reported in the Results section.

Finally, to evaluate the clinical sensitivity of the real-time PCR assays ART and CAP/CTM, a total of 20 patients receiving TVR-based triple therapy for chronic HCV genotype 1 infection, in whom viral breakthrough (VBT) or relapse was diagnosed, were enrolled. Patients were recruited at Ogaki Municipal Hospital (Gifu, Japan), Shin-Matsudo Central General Hospital (Chiba, Japan), Kurume University Hospital (Fukuoka, Japan), The Jikei University Kashiwa Hospital (Chiba, Japan), and Nagoya City University Hospital (Aichi, Japan) and started TVR-based triple therapy from November 2011 to May 2013. Three of the patients recruited at Nagoya City University Hospital were also in the previously described group of 25 patients.

### Therapeutic protocols

#### TVR-based triple therapy

After TVR (Telavic; Mitsubishi Tanabe Pharma, Osaka, Japan), Peg-IFN-α2b (PEG-Intron; MSD, Tokyo, Japan) and RBV (Rebetol; MSD) were administered for 12 weeks, Peg-IFN-α2b and RBV without TVR were administered for an additional 12 weeks. TVR (750 mg po) was administered every 8 hours after a meal, and dose reduction was performed if necessary. Peg-IFN-α2b (1.5 μg/kg subcut. injection) was administered once weekly. RBV (600–1,000 mg po based on body weight [600 mg for patients weighing <60 kg, 800 mg for patients weighing 60–80 kg, and 1,000 mg for patients weighing >80 kg]) was administered daily.

#### DCV/ASV dual therapy

DCV (Dacluinza; Bristol-Myers Squibb Company, Tokyo, Japan; 60 mg po) was administered once daily, and ASV (Sunbepra; Bristol-Myers Squibb Company; 100 mg po) was administered twice daily for 24 weeks.

### Definitions of response

Serum specimens from patients receiving TVR-based triple therapy were assayed at weeks 1, 2, 4, 8, 12, 16, 20, and 24 for HCV by real-time PCR. Serum specimens from patients receiving DCV/ASV dual therapy were assayed at weeks 2, 4, 8, 12, 16, 20, and 24 for HCV by real-time PCR. The routine measurements of HCV RNA are described in the following subsection entitled “Detection of HCV RNA in serum specimens”. SVR24 was defined as undetectable HCV RNA in a serum specimen taken 24 weeks after the end of treatment [18]. Relapse was defined as undetectable serum HCV RNA at the end of treatment and detectable serum HCV RNA at the week-24 post-treatment follow up [18]. VBT was defined as a confirmed-on-treatment increase in an HCV RNA level of 1 log_10_ higher than the nadir or greater than 100 IU/mL in patients with briefly undetectable serum HCV RNA by CAP/CTM.

### Detection of HCV RNA in serum specimens

Each serum specimen was frozen at -80°C within 2 hours after the sample was taken from the patient [19]. Two real-time PCR assays for HCV were available. Routine measurements of HCV RNA were performed by the Cobas AmpliPrep/Cobas TaqMan instruments (Roche Diagnostics K.K., Tokyo, Japan) at each of the participating hospitals. This study used the first and second generations of the CAP/CTM assays (CAP/CTM v1.0 and CAP/CTM v2.0, respectively). CAP/CTM v1.0 sometimes fails to detect RNA copies of HCV genotypes 2 and 4 in specimens that are found to have high levels of HCV RNA by other assays [13] [20] [21]. CAP/CTM v2.0 represents several changes, including the requirement of a lower volume of serum sample (650 μL vs 850 μL for CAP/CTM v1.0) and additional probes that improve the quantification accuracy of HCV genotypes 2 and 4 [22]. Both versions of the CAP/CTM assays are equally sensitive for detecting HCV genotype 1 [22]. Therefore, for this report on patients infected with HCV genotype 1, CAP/CTM v1.0 and v2.0 are described as CAP/CTM.

ART was performed at Mitsubishi Chemical Medience Corporation (Tokyo, Japan), instead of the participating hospitals. The reported lower limits of detection were 1.08 log_10_ IU/mL for ART [23] and 1.2 log_10_ IU/mL for CAP/CTM [22, 24].

### Definitions of positive predictive value (PPV) for SVR, negative predictive value (NPV) for non-SVR, and false omission rate (FOR)

The PPV for SVR was defined as the number of patients who achieved SVR divided by the number of patients whose samples were negative for HCV RNA by the assay. The NPV for non-SVR was defined as the number of patients who did not achieve SVR divided by the number of patients whose samples were positive for HCV RNA by the assay. The FOR was the complement of the NPV (FOR = 1-NPV). The FOR was defined as the number of patients who achieved SVR divided by the number of patients whose samples were positive for HCV RNA by the assay.

### Comparing the clinical sensitivity of ART and CAP/CTM for assessing HCV RNA in the sera of patients receiving TVR-based triple therapy with a diagnosis of VBT or relapse by CAP/CTM

As described previously in the Patient section, this evaluation recruited 20 patients independently from the preceding 25 patients who were treated by TVR-based triple therapy. VBT or relapse was diagnosed in these 20 patients based on the CAP/CTM assay. Sera collected at the points of disappearance and the points of reappearance of HCV RNA were assayed by CAP/CTM and ART. The point of disappearance was defined as the first time HCV RNA was not detected by CAP/CTM in patients receiving TVR-based triple therapy, and the point of reappearance was defined as the time when HCV was again detected by CAP/CTM, which was considered to be VBT or relapse.

### Comparing the analytical sensitivity of CAP/CTM and ART

The WHO International HCV RNA standard (code 06/102) was used to prepare HCV-RNA-positive specimens. The dilution medium consisted of pooled sera that were HCV RNA negative by both CAP/CTM and ART assays. Serum was collected from patients receiving TVR-based triple therapy at week 8 or 12 after the start of treatment, and pooled. Every patient who provided serum negative for HCV RNA achieved rapid virological response (RVR) in addition to SVR.

CAP/CTM and ART assays were tested on dilution panels, and the 95% hit rates of the assays were compared. The WHO International HCV RNA Standard was serially diluted using the dilution medium to the following concentrations and numbers of samples: 50 IU/mL (5 samples), 25 IU/mL (10 samples), 12.5 IU/mL (20 samples), 6.25 IU/mL (20 samples), and 3.125 IU/mL (15 samples). The dilution panels were prepared at Nagoya City University Hospital and stored at -80°C until analysis. Each concentration panel was tested separately in a single run. CAP/CTM and ART measurements of HCV RNA were performed at Nagoya City University Hospital and Mitsubishi Chemical Medience Corporation, respectively.

### Comparing the length of amplification products produced by ART and CAP/CTM

Both assays were performed at Nagoya City University Hospital, following the manufacturers’ protocols [13]; the assays were used to amplify HCV RNA in a serum specimen from a patient infected with HCV genotype 1. The amplification products obtained from ART and CAP/CTM, along with a 100-bp DNA ladder (Takara Bio Inc. Shiga, Japan), were electrophoresed on an agarose gel in 2% TRIS-borate-EDTA buffer at 50 V for 90 min. The gel was stained with ethidium bromide and viewed over an ultraviolet transilluminator.

### Cell transfection assay

Huh7.5.1 cells (provided by Dr. Frank Chisari) were grown in Gibco Dulbecco's Modified Eagle's Medium (DMEM) (Thermo Fisher Scientific Inc., Kanagawa, Japan) supplemented with 10% fetal bovine serum (FBS; Equitech-Bio, Kerrville, TX, USA), MEM non-essential amino acids (Invitrogen, Carlsbad, CA, USA), 100 units/mL penicillin (Wako Pure Chemical Industries, Ltd., Osaka, Japan), and 100 μg/mL streptomycin (Wako Pure Chemical Industries, Ltd.) at 37°C in a 5% CO_2_ incubator. The Huh7.5.1 cells were inoculated with an infectious JFH1 plasmid [25], as previously described. Briefly, 1×10^6^ Huh7.5.1 cells in 8 mL of the culture medium were seeded into 10-cm tissue culture dishes 1 day before infection. On the day of infection, the JFH1 plasmid was added to the cells at a multiplicity of infection of 0.03. The JFH1-infected cells were used for subsequent experiments.

The medium (2 mL), which contained 8×10^4^ JFH1-infected Huh7.5.1 cells, was added to each well of 6-well plates, and TVR was immediately added to a final concentration of 10 μM or TVR plus IFN (IFN-α, MSD, Tokyo, Japan) were added to final concentrations of 10 μM and 100 IU/mL, respectively. The JFH1-infected cells were passed, and at each passage (day 0, 4, 6, 10, 13, 17 and 19) supernatant was collected, filtered through a 0.45-μm filter, and stored frozen at -70°C until analysis. The specimens were thawed and HCV RNA and HCV antigen (HCV Ag) were assayed. Both CAP/CTM and ART were used for detection of HCV RNA, and HCV Ag was assessed by the Abbott Architect HCV Ag assay (Abbott Diagnostics, Lake Forest, IL, USA).

### Statistical analysis

To assess the clinical sensitivities of ART and CAP/CTM, categorical variables were compared by the Fisher exact test. The chi-square test was used to assess the analytical sensitivity of CAP/CTM and ART. *P* values less than 0.05 were regarded as statistically significant.

## Results

### HCV RNA levels of patients receiving TVR-based triple therapy at week 4

**Fig 1:**
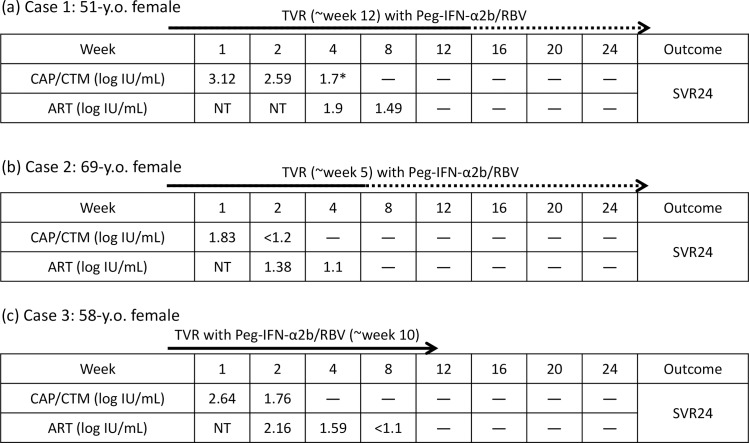
Typical cases receiving TVR-based triple therapy with HCV RNA undetectable by CAP/CTM and detectable by ART. These 3 patients were receiving TVR-based triple therapy and achieved SVR24. (a) Case 1 received TVR-based triple therapy for 12 weeks, and then the patient was treated without TVR up to week 24. HCV RNA was detected by ART only at week 8. *The CAP/CTM result was obtained at week 3. (b) Case 2 received TVR-based triple therapy for 5 weeks, and then the patient was treated without TVR up to week 24. HCV RNA was detected by ART only at week 4. (c) Case 3 received TVR-based triple therapy for 10 weeks, and HCV RNA was detected by ART only at weeks 4 and 8. y.o., year old.—, HCV RNA was undetected. NT, not tested.

The discordance between the results of ART and CAP/CTM is remarkable. Of the 25 serum samples collected from patients receiving TVR-based triple therapy, none of the samples were HCV RNA positive by CAP/CTM and HCV RNA negative by ART, and 10 samples were HCV RNA negative by both CAP/CTM and ART. In contrast, 13 samples were HCV RNA positive by ART and HCV RNA negative by CAP/CTM, and 2 samples were HCV RNA positive by both CAP/CTM and ART. Typical cases that were HCV RNA negative by CAP/CTM and HCV RNA positive by ART are shown as cases 1, 2, and 3 in Fig 1.

The majority of samples (13/25; 52%) were discordant (HCV-RNA negative by CAP/CTM and HCV-RNA positive by ART), and most of the discordant samples (11/13; 84.6%) were quantified by ART to be below the limit of detection (1.08 IU/mL). These results indicate that ART can detect HCV RNA with higher sensitivity than CAP/CTM in patients receiving TVR-based triple therapy.

The PPV for SVR and NPV for non-SVR as calculated based on the detection of HCV RNA by ART and CAP/CTM at week 4, are summarized in Table 1. Of 25 patients, 23 achieved SVR24; 1 was found to have relapse, and 1 was found to have VBT. The PPV was high in both assays (90% [9/10] and 91.3% [21/23]) for ART and CAP/CTM, respectively). The NPVs of ART and CAP/CTM were 6.7% (1/15) and 0% (0/2), respectively. In general, NPV was affected by the treatment outcomes in the study population. The SVR rate at 4 weeks in this study was too high (23/25; 92%) to use the NPV as an index of evaluation, because of the high SVR rate of patients with the *IL28B* rs8099917 TT allele who are treated by TVR-based triple therapy [26]. Notably, the FOR of ART was 93.3% (14/15). The majority of patients (14/15; 93.3%) who were HCV RNA positive by ART at week 4 were able to achieve SVR, indicating that ART at week 4 of treatment could not be used to predict outcome for more than half the patients (14/25; 56%).

**Table 1:** PPVs for SVR and NPVs for non-SVR in patients receiving TVR-based triple therapy at week 4, based on the results of CAP/CTM and ART assays for detecting HCV RNA.

	CAP/CTM		ART	
	PPV	NPV	PPV	NPV
Week 4	21/23	0/2	9/10	1/15
	(91.3)	(0)	(90)	(6.7)

Data are expressed as number (%).

### Clinical sensitivity of ART and CAP/CTM in patients receiving triple therapy, who were identified with relapse or VBT

There were 20 patients who were intentionally recruited from several hospitals for this evaluation. Other details about these patients were presented in the Methods. The diagnosis of VBT or relapse was performed based on the results by CAP/CTM.

**Fig 2:**
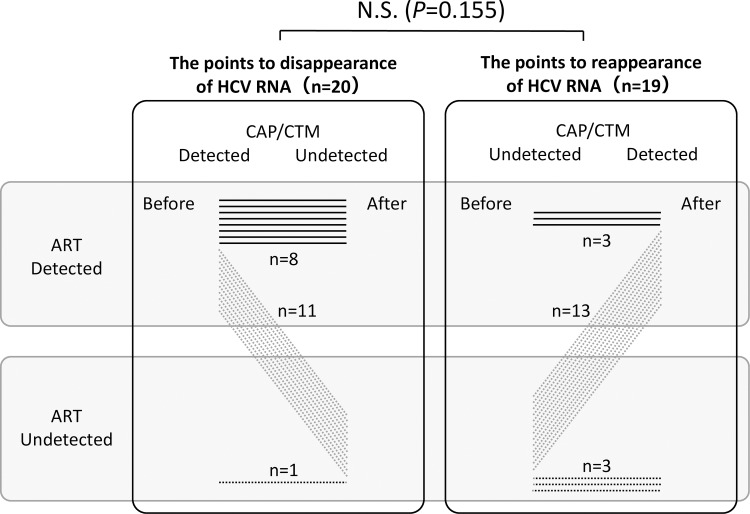
The points of disappearance and reappearance of HCV RNA by the CAP/CTM and ART assays of serum specimens from patients receiving TVR-based triple therapy, who were identified with relapse or VBT. Solid lines, CAP/CTM and ART obtained the same results. Dotted lines, CAP/CTM and ART obtained different results. N. S., not significant by chi-square test.

The times to disappearance of HCV RNA as determined by CAP/CTM are shown in Fig 2. ART and CAP/CTM yielded the same results for 11 (VBT 4, relapse 7) of 20 patients (55%). ART detected HCV RNA in 8 (VBT 3, relapse 5) of 20 patients (40%), whereas CAP/CTM did not. These results suggest that the times to disappearance of HCV RNA when the ART assay is used is longer than when the CAP/CTM is used to evaluate patients receiving TVR-based triple therapy who are identified with VBT or relapse.

The times to reappearance of HCV RNA determined by CAP/CTM are also shown in Fig 2. ART and CAP/CTM yielded the same results for 13 (VBT 5, relapse, 8) of 19 patients (72%). ART detected HCV RNA in 3 of 19 (16%) patients, who later received the diagnosis of relapse, when CAP/CTM did not detect HCV RNA. CAP/CTM detected HCV RNA in 3 other patients (16%), who were found to have VBT, when ART did not detect HCV. These results indicate that the times to reappearance of HCV RNA when ART is used are similar to the times of reappearance when CAP/CTM is used for assessing patients receiving TVR-based triple therapy in whom VBT or relapse is diagnosed. However, ART can detect HCV RNA for a longer time than CAP/CTM, when the level of HCV RNA is reduced by TVR-based triple therapy.

### Analytical sensitivity of ART and CAP/CTM

**Fig 3:**
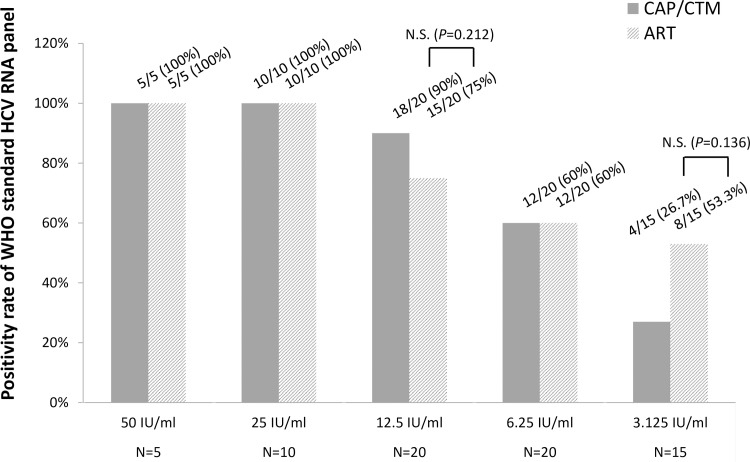
Dilutions of the WHO HCV RNA Standard that were assayed by CAP/CTM and ART. Positivity rates of the assays of replicate samples at each concentration are shown. N. S., not significant by the Fisher exact test.

The dilution medium consisted of a pool of sera from patients receiving TVR-based triple therapy, who were negative for HCV RNA by both CAP/CTM and ART. The results are shown in Fig 3. Both ART and CAP/CTM detected HCV RNA at concentrations of 50 and 25 IU/mL in all the tested replicates. At a concentration of 12.5 IU/mL, 18/20 (90%) and 15/20 (75%) of tested replicates were positive by CAP/CTM and ART, respectively (*P* = 0.212). At a concentration of 6.25 IU/mL, 12/20 (60%) of tested replicates were positive by both CAP/CTM and ART. At a concentration of 3.125 IU/ml, 4/15 (27%) and 8/15 (53%) of tested replicates were positive by CAP/CTM and ART, respectively (*P* = 0.136) (Fig 3). These results indicate that CAP/CTM and ART detect HCV RNA with similar sensitivity. In addition, the results suggest that the analytical sensitivity of CAP/CTM might not be reduced by the presence of TVR in the serum.

### Length of CAP/CTM and ART amplification products

**Fig 4:**
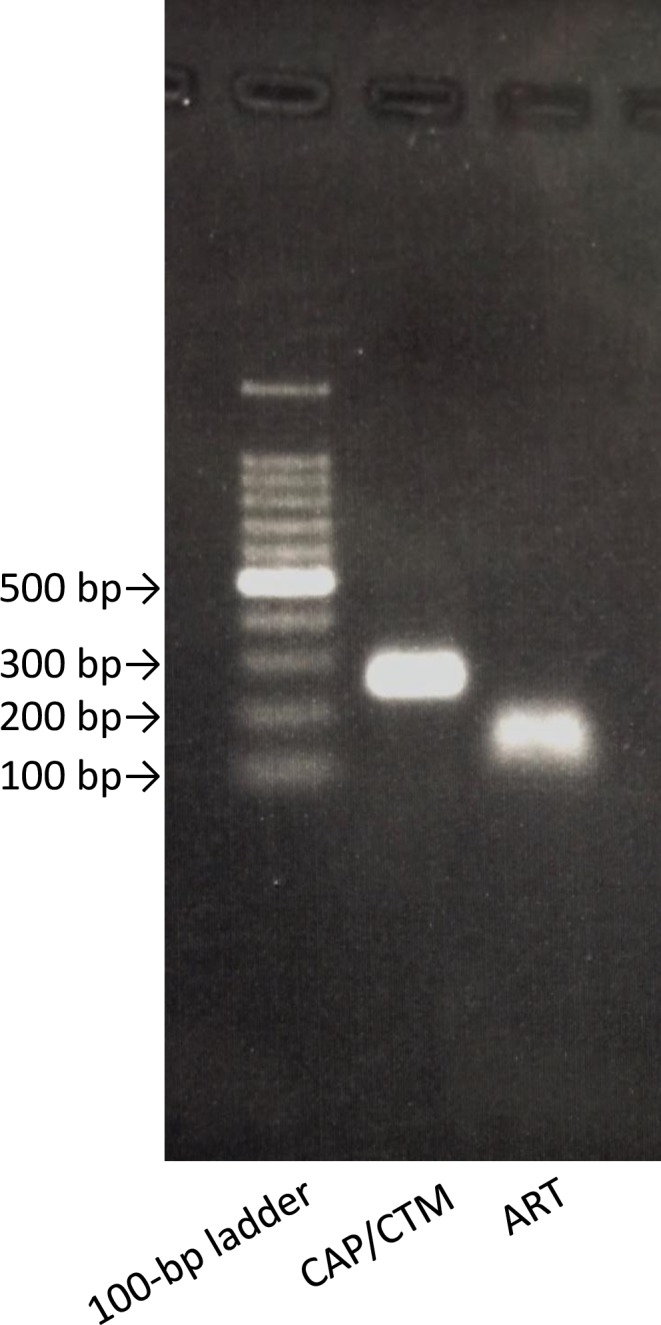
Amplification products produced by CAP/CTM and ART. Each lane from left to right shows the following: molecular weight marker (100-bp ladder), CAP/CTM amplification product, and ART amplification product.

The amplification products produced by CAP/CTM and ART are shown in Fig 4. The length of the CAP/CTM v1.0 amplification product was approximately 250 bps. The amplification product produced by CAP/CTM v1.0 has approximately the same length as the amplification product produced by CAP/CTM v2.0 (data not shown). The length of the ART amplification product was shorter than 150 bps. If HCV RNA fragments resulting from a protease inhibitor persist in serum specimens, they might be detected more efficiently by the ART assay than by the CAP/CTM assay.

### *In vitro* study of tissue culture cells transfected with an HCV genome in the presence of TVR or TVR plus IFN

**Fig 5:**
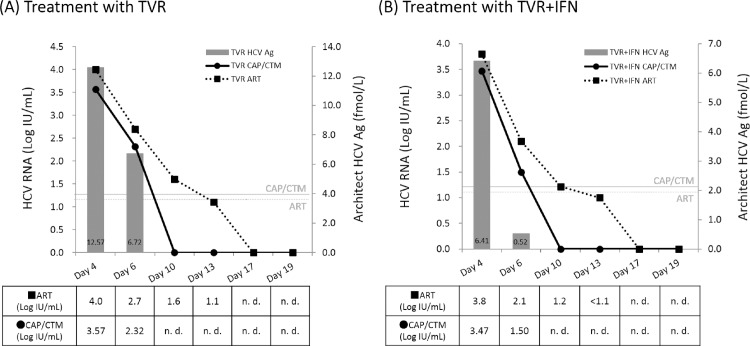
Typical results of *in vitro* cell transfection assay and detection of HCV RNA by CAP/CTM and ART. These experiments were performed twice, and the mean values of the 2 experiments are shown in Fig 5A and 5B. At day 13, ART detected HCV RNA below the reported lower limit of detection. (A) Results of cell cultures containing TVR 10 μM. (B) Results of cell cultures containing TVR 10 μM plus IFN 100 IU/mL. Bar graph, HCV Ag. Solid lines, CAP/CTM results. Dashed lines, ART results. n. d., not detected. Light-colored characters and lines show the lower limits of detection.

Typical results are shown in Fig 5. In the cell cultures containing TVR at 10 μM, CAP/CTM detected HCV RNA through day 6 and did not detect HCV RNA at day 10 and later. ART detected HCV RNA through day 13 and did not detect HCV RNA at day 17 and later. HCV Ag was detectable through day 6 and undetectable at day 10 and later (Fig 5A).

In the cell cultures containing 10 μM TVR plus 100 IU/mL IFN, CAP/CTM detected HCV RNA through day 6 and did not detect HCV RNA at day 10 and later. ART detected HCV RNA through day 10 in the detection range. At day 13, ART detected HCV RNA below the limit of detection (<1.08 Log IU/mL), but did not detect HCV RNA at day 17 and later. HCV Ag was detectable through day 6 and was undetectable at day 10 and later (Fig 5B). These results suggest that ART can detect HCV RNA more efficiently than CAP/CTM not only in serum but also in cell culture, implying that there were no substances from the human host in the sera that affected the sensitivity of CAP/CTM. Furthermore, it is likely that the presence of IFN in the cell cultures did not lead to decreased sensitivity of ART for HCV RNA. ART might detect HCV RNA fragments resulting from TVR treatment more efficiently than CAP/CTM, regardless of the presence of IFN.

### HCV RNA levels of patients receiving DCV/ASV dual therapy at weeks 4 and 8

The discordance between CAP/CTM and ART is notable. At week 8, of the 42 samples collected from patients receiving DCV/ASV dual therapy, no sample was HCV RNA positive by CAP/CTM and HCV RNA negative by ART, and 27 samples were HCV RNA negative by both CAP/CTM and ART. In contrast, 15 samples were HCV RNA positive by ART and HCV RNA negative by CAP/CTM, and no samples were HCV RNA positive by both CAP/CTM and ART. All the samples that were HCV RNA negative by CAP/CTM and HCV RNA positive by ART (15/42; 35.7%) were quantified by ART at levels below the limit of detection (1.08 IU/mL). In contrast, no samples were HCV RNA negative by ART and HCV RNA positive by CAP/CTM. As with TVR-based triple therapy, these results indicate that ART is more sensitive than CAP/CTM for detecting HCV RNA in serum samples from patients receiving DCV/ASV dual therapy.

For reference, assessments at week 4 in this group of patients did not reveal obvious differences at week 8 in the serum levels of HCV RNA determined by CAP/CTM and ART. Of the 40 samples, 15 samples (37.5%) were discordant (HCV-RNA negative by CAP/CTM and HCV-RNA positive by ART), suggesting that the discordance between the results of ART and CAP/CTM at week 4 is not as great, compared with that at week 8.

The PPV for SVR and NPV for non-SVR, which were calculated based on the results of CAP/CTM and ART quantification of HCV RNA in serum specimens from weeks 4 and 8 after the start of therapy, are summarized in Table 2. To compare the predictive accuracy of CAP/CTM and ART, we studied patients receiving DCV/ASV dual therapy at weeks 4 and 8. At week 4, of 40 patients, 34 patients achieved SVR24, 5 patients were identified with relapse, and 1 patient with VBT. Both assays had a high PPV; the PPVs of CAP/CTM and ART were 88.9% (24/27) and 100% (12/12), respectively. The NPV of CAP/CTM and ART were 23.1% (3/13) and 21.4% (6/28), respectively, suggesting that CAP/CTM and ART assessment of patients at week 4 of treatment did not reveal obvious differences that would affect the prediction of treatment outcome (Table 2). At week 8, of 42 patients, 34 patients achieved SVR24, 6 patients were identified with relapse, and 2 patients with VBT. Both assays had a high PPV; the PPVs of CAP/CTM and ART were 81.0% (34/42) and 85.2% (23/27), respectively. The NPV of ART was 26.7% (4/15). Notably, 15 of 42 patients (35.7%) were HCV RNA positive by ART, of which 11 patients (73.3%) achieved SVR (Table 2). The FOR of ART was 73.3% (11/15), indicating that ART assessment of patients at week 8 of treatment also does not accurately predict treatment outcome.

**Table 2:** The PPVs for SVR and NPVs for non-SVR in patients receiving DCV/ASV therapy, based on the results of CAP/CTM and ART assays for detecting HCV RNA in samples taken at weeks 4 and 8.

		CAP/CTM		ART	
		PPV	NPV	PPV	NPV
**Week 4**	All patients	24/27	3/13	12/12	6/28
	(n = 40)	(88.9)	(23.1)*^1^	(100)	(21.4)*^1^
	Treatment-naïve patients	11/12	1/8	5/5	2/15
	(n = 20)	(91.7)	(12.5)*^2^^,^ ^4^	(100)	(13.3)*^2^^,^ ^5^
	Treatment-experienced patients	13/15	2/5	7/7	4/13
	(n = 20)	(86.7)	(40.0)*^3^^,^ ^4^	(100)	(30.8)*^3^^,^ ^5^
**Week 8**	All patients	34/42	0/0	23/27	4/15
	(n = 42)	(81.0)	(0)	(85.2)	(26.7)
	Treatment-naïve patients	18/22	0/0	12/15	1/7
	(n = 22)	(81.8)	(0)	(80.0)	(14.3)*^6^
	Treatment-experienced patients	16/20	0/0	11/12	3/8
	(n = 20)	(80.0)	(0)	(91.7)	(37.5)*^6^

Data are expressed as number (%).

*^1^,*^2^, *^3^, *^4^, *^5^ and *^6^ Difference is not significant (*p* = 0.91, 0.63, 0.71, 0.64, 0.52 and 0.52, respectively).

To compare the predictive accuracy of CAP/CTM and ART at weeks 4 and 8 in treatment-naïve patients and treatment-experienced patients, these two groups were analyzed separately. The results are summarized in Table 2. The results suggested that CAP/CTM and ART assessment of patients at week 4 of treatment did not reveal obvious differences that would affect the prediction of treatment outcome in either the treatment-naïve or treatment-experienced patients. However, the results indicated that ART assessment at week 8 of treatment did not accurately predict treatment outcome in either the treatment-naïve or treatment-experienced patients (Table 2).

## Discussion

In this study, we determined the analytical and clinical sensitivities of two commercial real-time PCR assays, CAP/CTM and ART, for the detection of HCV RNA in patients receiving protease inhibitor-based therapy. Although several studies have already been published on the kinetics of HCV and comparisons of real-time PCR HCV assays used to assess patients receiving TVR-based triple therapy, to the best of our knowledge, this is the first study that compared real-time PCR HCV assays used to assess patients receiving DCV/ASV dual therapy.

In addition to determining the sensitivity of the two assays, the purposes of our study were to compare the efficacy of CAP/CTM and ART in patients receiving the protease inhibitor-based treatments of TVR-based triple therapy and DCV/ASV dual therapy, and to confirm that the sensitivity of ART for detecting HCV RNA was superior to that of CAP/CTM.

With respect to the clinical sensitivity of the assays for evaluating the sera of patients receiving TVR-based triple therapy who were found to have VBT or relapse, the times to disappearance of HCV RNA as determined by ART were longer than the times to disappearance by CAP/CTM. This result supports our third hypothesis, namely, that small noninfectious HCV RNA fragments remaining in the sera of patients who have resolved HCV viremia are easily detected by ART; which is further supported by the relative lengths of the ART and CAP/CTM amplification products (< 150 bps vs ~ 250 bps, respectively), as shown in Fig 4. In fact, previous reports have shown that infectious HCV RNA and small noninfectious HCV RNA can be delivered by exosomes, because exosomes can protect naked RNAs such as noninfectious HCV RNA fragments from RNase, which is present in all bodily fluids [27–29]. Furthermore, exosomes containing intact HCV RNA fragments should be stable in serum over time, and of the two assays, only ART would be able to detect small HCV RNA fragments in serum exosomes. Our hypothesis is considered to be also supported by the results of the cell transfection experiment (Fig 5), and our in vivo and in vitro results might imply that HCV RNA becomes fragmented by TVR treatment in both cell culture and serum.

HCV Ag can be used as an indirect marker of HCV replication [30]. The results of the cell transfection experiment suggested that the kinetics of HCV Ag levels were more strongly correlated with CAP/CTM than with ART. Therefore, we speculate that the results of ART assays reflect not only the levels of viable HCV but also the levels of HCV fragments. Further study will be needed to confirm it.

With respect to the analytical sensitivity of the assays, both CAP/CTM and ART showed similar sensitivity for detecting HCV RNA in dilutions of pooled sera containing TVR. There was no possibility that the sensitivity of CAP/CTM was inhibited by the diluent. This result does not support the other 2 hypotheses; CAP/CTM and ART detected HCV RNA with comparable analytical sensitivity.

DCV/ASV dual therapy has been available in Japan for patients with HCV genotype 1 infection since September 2014. Sofosbuvir (Sovaldi; Gilead Sciences, Inc., Tokyo, Japan), which should be used in combination with RBV, has been available for patients with HCV genotype 2 infection since July 2015. Ledipasvir/sofosbuvir (Harvoni; Gilead Sciences, Inc.) has been available for patients with HCV genotype 1 infection in Japan since September 2015. Although DCV/ASV dual therapy has not been approved to treat HCV infection everywhere in the world, and ledipasvir/sofosbuvir is the first-line therapy, DCV/ASV dual therapy has clinical importance in Asian countries such as Japan, China, Korea, and Taiwan. In Japan, DCV/ASV dual therapy is expected to be cost-saving for patients with HCV genotype 1b who have failed prior therapy or are IFN-ineligible/intolerant [31]. In addition, DCV/ASV dual therapy for chronic hemodialysis patients with HCV infection is highly effective and well tolerated, even for elderly patients and patients with liver cirrhosis or the resistance-associated variant NS5A-Y93H at baseline [32]. Differences in the mechanisms of action of protease inhibitors against HCV RNA are minimal. To determine whether or not the sensitivities of CAP/CTM and ART are different, we investigated an IFN-free regimen that included a protease inhibitor (ASV). ART assays of serum specimens from patients treated with therapy based on protease inhibitors may not provide accurate quantitative HCV RNA results that reflect infectious virus. In our study, we confirmed that although ART detected HCV RNA in the serum specimens of 15 patients at week 8, eleven of these patients were able to achieve SVR. That is, the FOR of ART was 73.3% (11/15), which suggests that detection of HCV RNA at week 8 does not accurately predict treatment outcomes.

This study has limitations. First, TVR-based triple therapy was indicated only for patients infected with HCV genotype 1b with the *IL28B* rs8099917 TT allele, whose outcomes were predicted as SVR [8]. Therefore, TVR-based triple therapy for these patients was quite effective. Because most of the patients achieved SVR, comparing the PPVs for SVR and the NPVs for non-SVR of ART and CAP/CTM was difficult. Second, for the patients who received TVR-based triple therapy and were enrolled because they received a diagnosis of VBT or relapse, the diagnoses were only decided by the CAP/CTM assay, not by the ART assay. As a result, HCV RNA was undetectable by CAP/CTM at the disappearance points, and was detected by CAP/CTM at the reappearance points. Third, although among NS3 protease inhibitors, simeprevir is surely a global standard, the discordant results provided by CAP/CTM and ART that have been observed in patients treated with telaprevir-based triple therapy, have not yet been reported for samples from patients treated by simeprevir. Further study is needed to determine if the same phenomenon would occur with simeprevir. Finally, the times during treatment that the assays were used to evaluate the serum levels of HCV RNA were different for the 2 treatments regimens. The serum HCV RNA levels of the patients receiving TVR-based triple therapy were assessed at week 4 after initiation of therapy.

In conclusion, although the CAP/CTM and ART assays detect HCV RNA with comparable analytical sensitivity, CAP/CTM might be the preferred assay for predicting the clinical outcomes of patients receiving protease inhibitor-based therapy. ART can detect not only HCV RNA that reflects infectious virus, but also small nonviable HCV RNA fragments. Additional confirmatory studies that compare these assays for monitoring patients receiving sofosbuvir-based therapy are warranted.
